# Development and validation of the Nutrition Care Process Assessment of Brief Level of Expertise screening tool

**DOI:** 10.3389/fnut.2025.1572181

**Published:** 2025-05-02

**Authors:** Luc R. LaBonte, Constantina Papoutsakis, Sherri Lewis, Casey Colin

**Affiliations:** ^1^Department of Nutrition Science, East Carolina University, Greenville, NC, United States; ^2^Department of Nutrition and Dietetics, University of North Florida, Jacksonville, FL, United States; ^3^Academy of Nutrition and Dietetics, Chicago, IL, United States; ^4^James A. Haley Veterans’ Hospital, Tampa, FL, United States

**Keywords:** Nutrition Care Process, validation, proficiency, nutrition education, questionnaire development, screening

## Abstract

**Objective:**

To develop and validate an abbreviated screening tool to screen Nutrition Care Process (NCP) proficiency.

**Methods:**

The questionnaire was developed using existing literature. All iterations were reviewed by subject matter experts. The questionnaire underwent several methods of testing, including content validity, face validity, internal consistency reliability, and test–retest reliability. Questions were scored based on answer selection, and participants were categorized by observed levels of proficiency.

**Results:**

Internal consistency reliability testing indicated removal of two items, creating a 3-item questionnaire (the NCP Assessment of Brief Level of Expertise, or NCP-ABLE). All items met content (S-CVI = 0.94) and face (S-FVI = 0.94) validity and internal consistency (*α* = 0.75) and test–retest (*r* = 0.8, *p* = 0.009, 95% CI: 0.274, 0.962) reliability thresholds. Six (85.7%) of the subject matter experts reported higher degrees of proficiency with scores of 3 (highest quartile placement), whereas one expert demonstrated lower levels of proficiency through the score of 1 (second quartile placement).

**Conclusion:**

The NCP-ABLE met the established validity and reliability thresholds. This supports its utilization as a screen for NCP proficiency, particularly to identify individuals demonstrating lower levels of NCP knowledge proficiency. The NCP-ABLE may be effective for the screening of clinicians, educators, preceptors, and students for educational intervention or quality improvement initiatives. Future investigations may aim to validate the NCP-ABLE in other languages. Further research is needed to determine the relationship between NCP-ABLE scores and NCP implementation, possibly by comparing NCP-ABLE results and scores from robust assessments of NCP practice.

## Introduction

Since the adoption of the Nutrition Care Process (NCP) by the Academy of Nutrition and Dietetics in 2003, the NCP has continued to evolve (1–4). The 4-step model (assessment and reassessment, diagnosis, intervention, monitoring, and evaluation), composed of six clinical judgments across two care phases (problem identification and problem solving), has become a practice standard internationally (4). Among the benefits suggested by Splett and Myers (5) in their original proposal are improvements in consistency and quality of care. These advantages have been partially credited to the structure and guidance provided by NCP chains, or individual relationships between each of the clinical judgments (evidence, diagnosis, etiology, goal, intervention, and outcome) (6). The continued development of the NCP and advancements in informatics, terminology (in the form of the Nutrition Care Process Terminology, or NCPT), and data management methodology have established the theory behind the model’s use (4, 7).

While sound in theory, years would pass before sufficient evidence for NCP implementation would surface. Investigation teams led by Lewis et al. (8) and Mujlli et al. (9) would take advantage of an NCP chart audit tool (known as the Diet-NCP-Audit) developed by Lövestam et al. (10) to support NCP use. In one Veterans Health Administration facility, Lewis et al. (8) observed a 38% increased odds of problem resolution for every point increase on the overall audit score (scaling from 0 to 26). Furthermore, findings suggested that the presence of the etiology-intervention chain link in documentation elicited a 51 times higher problem improvement rate (8).

At the same time, Mujlli et al. (9) were conducting a similar study in Saudi Arabia with the aim of evaluating the impact of RDN NCP/T use and documentation on non-alcoholic fatty liver disease and metabolic syndrome in a pediatric population. Significant inverse correlations with audit score in respect to changes in body mass index from the 6-to-12-month follow-up period and alkaline phosphatase measurements were observed (9). Despite the usage of NCP in different countries and under dissimilar context, improved Diet-NCP-Audit scores were found to be significantly associated with improved patient outcomes (8, 9).

Colin et al. (11) continued to expand the field with a secondary analysis of the 2017–2019 diabetes registry cases compiled via The Academy of Nutrition and Dietetics Health Informatics Infrastructure (ANDHII) Dietetics Outcome Registry (DOR). The DOR, one of the prominent tools available in ANDHII, allows for aggregation of de-identified encounter data submitted by nutrition and dietetics professionals (12). While the linkage associated with problem resolution varied from previous investigations, the NCP implementation in the presence of the evidence-diagnosis link was a significant predictor of diagnosis resolution. These findings were among the most notable to support the effective use of NCP/T (and its underlying clinical judgments as measured by chain links) for the purpose of improvements in quality of nutrition documentation.

The body of literature suggests that increased NCP knowledge and quality implementation are associated with greater improvements in the original NCP benefits introduced by Splett and Myers (5). Despite preceding these findings, the Academy of Nutrition and Dietetics Practice paper (13) discussing analytical skills needed for effective nutrition assessment and diagnosis was among the first works describing the characteristics associated with NCP proficiency. This was later captured in the first NCP update paper (3), which suggests that proficiency involves thorough understanding of the model (and underlying critical judgments), effective organization of data, prioritization of actionable problems, and recognition of patterns between care-related constructs. The qualities expressed at the level of proficiency are particularly essential in the problem identification phase, which involves critically evaluating nutrition assessment findings to identify the presence of nutrition problems and their most influential etiologies (2, 6, 14). This surpasses prerequisite stages of understanding that focus on recognition of model steps, ability to structure care, selection of assessment tools and procedures, and differentiation of problems, etiologies, and symptomologies (3, 13). Collectively, NCP proficiency can be described as having theoretical knowledge that exceeds competency and allows for higher-level nutrition assessment and diagnosis (including recognition of patterns between such as problems, etiologies, and symptomologies) and prioritization of actionable nutrition interventions.

The most widely practiced form of individual NCP assessment involves a retrospective approach using documentation audit tools. Tools such as the aforementioned Diet-NCP-Audit and the NCP Quality Evaluation and Standardization Tool (NCP QUEST) can effectively evaluate NCP implementation (10, 15). NCP QUEST in particular has been endorsed for its high content validity and potential for teaching and elevating outcomes management (15); however, chart auditing methods may introduce additional workloads for managers that are unfamiliar with or disinterested in these tools. These audits are also retrospective in nature, meaning that their items cannot be proactively administered in advance of care encounters unless used in resource-demanding educational simulations. A prospective approach in the form of knowledge screens may yield immediate data to recognize individuals or populations at risk of low NCP knowledge, allowing for educational intervention, quality improvement, and investigatory purposes. Assessments of comprehension have been developed, such as the NCP knowledge quiz in Module 4 of the International Nutrition Care Process Implementation Survey (INIS) (16). While being recognized as the most comprehensive web-based instrument for evaluation of NCP knowledge, attitudes, perceptions, and implementation, the questions introduced focus on basic NCP knowledge scaled to levels below proficiency (16). To the knowledge of the authors, a concise tool for evaluating the level of proficiency had not been validated. The purpose of this investigation was to develop and validate an electronic questionnaire to screen NCP proficiency in nutrition and dietetics professionals.

## Methods

### Questionnaire development

The authors aimed to include no more than five items in the final revision for concision. Four fully original items (items 1, 2, 3, and 5) were developed from key points identified in literature. The NCP update papers (3, 4) were reviewed as a broad foundation for question development. The works from Charney and Peterson (13) and Thompson et al. (6) were reviewed for improved context, as these support the qualities stated by Swan et al. (3) Another item (item 4) was modeled on the concept introduced by the eighth item within the NCP Knowledge Quiz of the INIS (16) and reworded for context. The draft instrument was developed by the PI with subsequent review by the authors for revision. Formal assessment, involving multiple rounds of adjustment, followed with the assistance of seven subject matter experts. Qualtrics, version March 2024 (Qualtrics) (17) hosted the survey for the investigation team and disseminated iterations to experts. Each round of revision included evaluation of expert feedback, reliability and validity testing, and discussion within the authors (Table 1).

**Table 1:** Validation methodology.

Type of validation	Validation methods
Content validity	Individual item relevancy assessment using a four-point scale (1—irrelevant, 2—somewhat relevant, 3—relevant, 4—very relevant); acceptability threshold set at 0.7 for each item (I-CVI) and the instrument (S-CVI).
Face validity	Binary response of “Yes” or “No” to assess the acceptability of each item; agreement for each item (I-FVI) and instrument (S-FVI) to assess interrater agreement with acceptability threshold at 0.8.
Internal consistency reliability	Cronbach’s α coefficient from expert participants with an acceptability threshold set at 0.7.
Test–retest reliability	ICC calculated from chosen sample between two time points; acceptability threshold set at 0.7.

I-CVI, individual item content validity index; I-FVI, individual item face validity index; ICC, intraclass correlation; S-CVI, content validity index for all items; S-FVI, face validity index for all items.

### Subject matter expert criteria

Eligibility for experts included maintenance of registered dietitian nutritionist (RDN) registration through the Commission on Dietetic Registration, self-perception of NCP proficiency, and at least 3 years of practice- or education-based NCP usage. Expert recruitment involved convenience sampling, as 11 eligible peers of the investigation team across academic, clinical, and research institutions were invited to participate in validation. After confirming eligibility and interest, prospective experts received an email containing the background information on the study, a copy of the draft instrument, and an invitation link to participate in a modified copy of the survey for assessment.

### Content validity

The draft questions approved by the authors underwent content validity assessments endorsed by authors from the nursing discipline (18, 19). After each draft question was answered by subject matter experts, an item assessing relevancy using a four-point Likert scale (one being irrelevant and four being extremely relevant) followed (18). Upon completion of each question and prompt, a text box was provided for anonymous feedback. Each item receiving a score of three or four on the Likert scale yielded one point, with a score of one or two receiving zero points. Content validity index (CVI) was calculated for each individual question (I-CVI) and the entire instrument (S-CVI). All items with an I-CVI of less than 0.7 were identified for revision. A lower threshold of 0.7 was implemented due to broadness and concision of the questionnaire, as values less than 0.7 necessitate removal (18, 20). Feedback provided for these items and the collective instrument was considered, and experts were asked to repeat the process for the newly revised questions.

### Face validity

Face validity was assessed with the goal of ensuring that the items are in alignment with research aims. The experts were determined to be an appropriate sample for this assessment, as they represent the target audience and provide perspective from the expert and participant perspectives. A binary selection (“Yes” or “No”) followed each individual content validity item, requesting each expert to indicate their agreement on the question’s appropriateness for the intended aims. Disagreeable experts were encouraged to leave feedback in the text box mentioned during content validity assessment. Interrater agreement scores, as face validity indexes (FVI) were calculated for each item (I-FVI) as well as an average for the entirety of the block (S-FVI) with an acceptability threshold of 0.8 (indicating acceptable scale agreement) (21). Agreement below 0.8 indicated revision based on the feedback provided.

### Internal consistency reliability

Internal consistency reliability evaluation was conducted after content validity. Each item in this block was measured against the remaining individually using Cronbach’s *α* (22). Questions presenting a score less than 0.7 were considered unreliable and identified for revision (22). Feedback provided during the open-text portion of each item was considered. Revisions to or removal of identified questions were considered outcomes, with the latter potentially resulting in the generation of a new item for assessment. These were considered when administering the survey in following iterations.

### Test–retest reliability

Upon conclusion of the last satisfactory distribution for content validity, face validity, and internal consistency reliability, item scores were collected. Subject matter experts were encouraged to provide candid answers with each attempt to ensure that the first point for test–retest reliability assessment was valid. Retest items were distributed 1 week after each item passed previous validity and reliability assessment and remained available for 1 week. Intraclass correlation coefficients (ICC) were calculated from total scores (22). Assessments yielding a *r* value less than 0.7 would be considered unreliable and indicate revision (22).

### Scoring

A proficiency score (PS) was assigned to the first attempt based on item responses. A correct response awarded one point, and all incorrect responses yielded an item score of zero. Participants were categorized based on the total number of correct answers, with proficiency categories equal to the total number of included items in addition to a category for no correct answer selection (or score of 0). Higher scores indicated a stronger degree of proficiency, whereas lower scores suggested lesser levels of proficiency. Table 2 lists the scoring stratification descriptions.

**Table 2:** Proposed scoring stratification.

Instrument score	Scoring description
0	Suggestive of non-proficiency; indication for general competency assessment
1	Suggestive of a low level of proficiency; potentially beginning to develop knowledge beyond competency
2	Suggestive of a mid-level of proficiency; knowledge more consistent with a practitioner at the level of proficiency
3	Suggestive of a high level of proficiency; indication for Advanced Practice/Expert assessment

## Results

### Subject matter experts

The five-item draft was accepted by the investigation team to undergo the following testing. Nine of the 11 (81.8%) solicited peers accepted the invitation with seven (63.6%) completing all subsequent steps of the validation process. Three of the seven (42.8%) participants held terminal degrees in dietetics, and all but one of the experts (14.3%) have experience as educators or preceptors in Accreditation Council for Nutrition and Dietetics (ACEND)-accredited programs.

### Validity and reliability testing

The original 5-item questionnaire with validation items underwent three rounds of revision. Four (80%) of the items (1, 2, 3, and 5) passed acceptability thresholds for content and face validity. The third iteration of item 4 met the threshold. All five items met test–retest reliability thresholds; however, the inclusion of items 1 and 5 weakened the collective internal consistency reliability. Removal of these items increased Cronbach’s alpha to 0.75 and met the established threshold (Table 3). The final iteration of the proficiency questionnaire containing items 2, 3, and 4, referred to as the Nutrition Care Process Assessment of Brief Level of Expertise (NCP-ABLE), received no further feedback from the investigation team (Table 4).

**Table 3:** NCP-ABLE final iteration reliability and validity results.

Construct	Measure	Value (s)
Content validity index	S-CVI	0.905
Face validity index	S-FVI	0.905
Internal consistency reliability	Cronbach’s α	0.75
Test–retest reliability	ICC	*r* = 0.8, *p* = 0.009, 95% CI (0.274, 0.962)

CI, confidence interval; ICC, intraclass correlation coefficient; NCP-ABLE, Nutrition Care Process Assessment of Brief Level of Expertise; S-CVI, content validity index for all items; S-FVI, face validity index for all items.

**Table 4:** NCP-ABLE items.

Item	Options (score)
1: What is at the center of the Nutrition Care Process Model?^*^	The context of care that influences how consumers receive nutrition information (0)
	The skills and knowledge that are unique to nutrition professionals (0)
	The interactions between recipients of care and the nutrition professional (1)
	The triggering event that initiates the need for assessment (0)
	I do not know or am unsure (0)
2: By theory, proper implementation of the NCP/T should result in a reduction of which of the following care-related aspects?	Consistency (0)
	Autonomy (0)
	Patient-centeredness (0)
	Ambiguity (1)
	I do not know or am unsure (0)
3: Which of the following is the first concept in the NCP chain?	Evidence (1)
	Intervention (0)
	Goal (0)
	Etiology (0)
	I do not know or am unsure (0)
4: As a monitoring indicator, documenting changes in patient/client weight would be best placed in which of the following categories during development of a PES statement?	Problem (0)
	Diagnosis (0)
	Signs and Symptoms (1)
	Etiology (0)
	I do not know or am unsure (0)
5: Whenever possible, it is considered best practices to target a nutrition intervention at which of the following?^*^	The problem’s nutrition diagnostic term (1)
	The signs and symptoms associated with the problem (1)
	The medical diagnosis (1)
	The etiology of the problem (1)
	I do not know or am unsure (0)

^*^Removed from final iteration of the instrument. NCP, Nutrition Care Process; NCP-ABLE, Nutrition Care Process Assessment of Brief Level of Expertise; NCP/T, Nutrition Care Process Terminology; PES, Problem-Etiology-Signs and Symptoms.

### Subject matter expert scoring

The distribution of collected scores was reviewed. When stratified in quartiles by score (0–3), six of the experts (85.7%) scored three points and displayed high levels of proficiency. One expert (14.3%) scored one point, suggesting a low degree of proficiency.

## Discussion

As the use of the NCP and NCPT becomes more widely accepted and supported for clinical outcomes, a focus on practitioner skill and quality implementation needs investigation. This focus is accomplished through chart evaluation using tools such as the NCP QUEST (15) and the Diet-NCP-Audit (10). These instruments are effective at evaluating NCP implementation and can be used for education (from peer-to-peer or clinician-to-student views) (15); however, their implementation may be seen as taxing by stakeholders and unnecessary by those with poorer NCP attitudes or reduced NCP knowledge. These, in addition to the retrospective nature of chart reviews, may serve as barriers to timely assessment of clinician performance and care outcomes. Since these tools assess documentation of the care provided, as opposed to measure of prerequisite NCP knowledge or skills, their items are difficult to administer in advance of care provision. Development of prospective predictors of NCP implementation quality and care outcomes through screening tools like NCP-ABLE has the potential to minimize barriers and augment current practices.

NCP-ABLE has demonstrated potential as a screening questionnaire and in particular to identify low collective NCP proficiency. This purpose may be markedly useful for various populations, including educators and clinicians. Those involved in education may see value in administering NCP-ABLE to students at various points in their pre-registration preparation, such as following completion of didactic programming or preceding clinical supervised practice. Likewise, educational administration can implement NCP-ABLE to identify support needs for active preceptors. A clinical preceptor with low NCP proficiency may require educational intervention to become more familiar with current practice expectations before providing practice-based education and overseeing interns’ tasks. Moreover, clinical nutrition managers may use NCP-ABLE to screen current and future RDNs being supervised. This is especially apparent to support improvements in quality of care, as the recent literature strongly supports the relationship between NCP implementation and care outcomes (8, 9, 11). Low proficiency scores may suggest poorer implementation, necessitating in-depth chart audits using NCP QUEST (15) or educational intervention.

A key consideration of this investigation involved defining NCP proficiency. To ensure that the intended construct was being measured, the NCP update papers (3, 4) served as the basis for item development since they express the formally accepted working qualities for NCP proficiency. The works from Charney and Peterson (13) and Thompson et al. (6) allowed for further conceptualization of questions since these were credited by the update papers (3, 4) when describing critical thinking qualities necessary for proficiency. Furthermore, the findings from Lewis et al. (8) and Colin et al. (11) suggest that links between evidence and diagnosis and etiology and intervention (both associated with proficiency) are crucial for problem resolution. These assumptions related to the NCP chain described by Thompson et al. (6) along with the validated items developed by Lövestam et al. (16) for the INIS, support the NCP-ABLE items’ relationship to proficiency. This literature collectively suggests that a relationship between NCP proficiency and care outcomes exists, and more sophisticated prospective tools must continue to be developed.

Despite the theoretical basis for the instrument’s development, limitations of this investigation must be addressed. One notable consideration from this investigation relates to the experts involved. Four (36.4%) had declined participation or did not complete the entirety of the process. This dropout was double what had been anticipated. Additionally, one expert scored in the second quartile of the instrument and may not have similar proficiency as their peers. Regardless, the minimum number of experts was still met per the protocol suggested by Rubio and colleagues (23) for the validation of similar constructs. The use of experts for test–retest reliability is recognized as a limitation. A sample of dietetics practitioners unfamiliar with NCP-ABLE would have been better indicated; however, the expert sample was utilized for prompt validation (allowing for timely pilot deployment as part of a more robust questionnaire).

Another possible weakness may be perceived as the number of items in the NCP-ABLE. Since the aim of the investigation was directed at creating a minimal burden screen (serving as a complement to other instruments) to broadly screen for proficiency, the concision of the NCP-ABLE was desirable. While originally developed to include five items, the final iteration totaled three. While this may reduce scoring variability and survey fatigue among participants, a decrease in extensiveness may reduce content validity and lack the ability to thoroughly identify individual NCP/T deficiencies. Each question, while focusing on a different aspect of NCP/T proficiency, may provide an indication of deficiency but cannot confidently determine this in isolation. Furthermore, subsequent administration of this short questionnaire to the same population may introduce a degree of learning bias and overestimate practitioner knowledge. This suggests that short-duration follow-up screening may be contraindicated.

Future investigations are needed to support its use for prediction of proficient NCP application. Experimentally comparing NCP-ABLE results against robust practice-based assessments that may measure care outcomes (such as the NCP QUEST) may further validate its use across settings (15). This proposed investigation can assess the accuracy of the instrument and provide data to support the use of NCP-ABLE items in questionnaires that predict general categorization of NCP knowledge. Furthermore, continued validation across various dietetics populations (including those practicing with different languages or in countries) is indicated to ensure validity and widespread usage.

## Conclusion

The NCP-ABLE met the established validity and reliability thresholds, suggesting appropriateness for its use as a screening tool of NCP knowledge proficiency. Various populations, such as educators and clinical nutrition managers, may find value from its screening potential to identify individuals with low collective NCP proficiency. While this investigation does not support its use as a predictor of NCP implementation quality or patient outcomes, future studies comparing the NCP-ABLE against more rigorous assessment methods that directly measure implementation may support broader use or revision of NCP-ABLE in its current iteration.

## Data Availability

The raw data supporting the conclusions of this article will be made available by the authors, without undue reservation.
